# Emotional security in an urban environment: The role of Design and Landscape

**DOI:** 10.1192/j.eurpsy.2026.11323

**Published:** 2026-07-31

**Authors:** N. Bouayed Abdelmoula, E. Abdelmoula

**Affiliations:** 1 LR23ES07 Genomics of Signalopathies at the service of Precision Medicine, Medical University of Sfax, Sfax; 2 LR21ES19 UMRAN, National School of Architecture and Urbanism of Tunis; 3 Doctoral school of architectural sciences and engineering (ED-SIA), Carthage University, Carthage, Tunisia

## Abstract

**Introduction:**

Emotional security in cities is a fundamental aspect of the well-being of residents, influenced not only by real security but also by perceived security and social belonging. Urban design and landscape architecture are essential to shape these factors.

**Objectives:**

The aim of this study is to outline the impact of urban design principles and landscaping interventions on emotional security in urban environments.

**Methods:**

We conducted a comprehensive review of the scientific literature using Web databases with the following keywords: urban design, urban landscape, architecture, security, emotions and well-being.

**Results:**

Our research found that well-designed urban spaces with clear lanes, adequate lighting, visibility and maintained landscapes reduce the fear of crime and increase the sense of security. Open and neglected green spaces encourage community engagement and promote a sense of belonging, which alleviates alienation and anxiety. The integration of social and environmental elements through participatory design and maintenance further strengthens the psychological comfort and emotional safety of residents.

**Conclusions:**

The interaction between the architectural layout, landscape features and community participation creates environments that enhance real and perceived security. Addressing only physical security is insufficient; supporting social cohesion and environmental quality is imperative for holistic emotional well-being. Thoughtful urban design and landscape planning play a central role in promoting emotional security in cities by natural supervision, accessibility, reducing fear, encouraging social interaction and environmental relaxation and promoting the psychological comfort of residents. Integrating these elements into urban planning can improve the quality of urban life and the resilience of communities.

**Disclosure of Interest:**

None Declared

